# Long-Term Outcome After Mustard Repair at Young Age: Longitudinal Follow-Up Into the Fifth Decade After Surgery

**DOI:** 10.1016/j.jacadv.2025.101984

**Published:** 2025-07-11

**Authors:** Sahra Ünlütürk, Robert M. Kauling, Judith A.A.E. Cuypers, Annemien E. van den Bosch, Alexander Hirsch, Mieke M.P. Driessen, Chiara Pelosi, Daniel J. Bowen, Ad J.J.C. Bogers, Willem A. Helbing, Isabella Kardys, Jolien W. Roos-Hesselink

**Affiliations:** a Department of Cardiology, Cardiovascular Institute, Thorax Center, Erasmus Medical Center, Rotterdam, the Netherlands; b European Reference Network for Rare, Low Prevalence and Complex Diseases of the Heart (ERN GUARD-Heart), Amsterdam, The Netherlands; c Department of Radiology and Nuclear Medicine, Erasmus Medical Center, Rotterdam, the Netherlands; d Department of Cardiothoracic Surgery, Thorax Center, Erasmus Medical Center, Rotterdam, the Netherlands; e Department of Paediatrics, Division of Cardiology, Erasmus Medical Center, Rotterdam, the Netherlands; f Clinical Epidemiology and Innovation Unit, Department of Cardiology, Erasmus Medical Center, Rotterdam, the Netherlands

**Keywords:** cardiac surgery, (dextro-/isolated/complete) transposition of the great arteries, long-term outcome, mustard repair, quality of life, systemic right ventricle

## Abstract

**Background:**

Concerns remain regarding the high incidence of morbidity in transposition of the great arteries (TGA) patients who underwent Mustard repair.

**Objectives:**

The purpose of this study was to evaluate the long-term outcome and quality of life of patients with TGA up to 48 years.

**Methods:**

Single-center, longitudinal cohort study evaluating consecutive patients with TGA who underwent Mustard repair between 1973 and 1980 at young age with extensive cardiac and subjective quality of life evaluation every decade.

**Results:**

Of the original cohort of 91 patients, 31 died and 2 underwent heart transplantation. Cumulative survival at 48 years was 61% (51% to 73%), with a shift in cause of death from sudden death to heart failure (HF) after 35 years of follow-up. Of the 44 eligible survivors, 34 (77%) were evaluated (71% male, median age 46 years) with a median follow-up of 46 (range: 40-48) years. Event-free survival was 14%, with cardiac reintervention (39%), symptomatic arrhythmia (39%), and HF (37%) being the most common events. Systemic right ventricular function (sRVF) declined over time, with 73% having an ejection fraction below 40%, and 95% having a reduced VO_2_max. Prolonged QRS duration was found to be a predictor for HF, early postoperative arrhythmias for late symptomatic arrhythmias, and a reduced sRVF for mortality. Self-perceived quality of life was good and remained stable over time.

**Conclusions:**

Mortality and morbidity after Mustard repair are substantial, with an event-free survival of only 14% at 48 years. The clinical condition deteriorated over time, necessitating timely consideration of advanced treatment options.

Transposition of the great arteries (TGA) is characterized by ventriculoarterial discordance with an estimated birth prevalence of 0.31 per 1,000 livebirths.^1^ Initially, only palliative procedures were possible. In the past decades, numerous advancements were made in cardiothoracic surgery, including the introduction of the atrial switch procedure, first by Senning in 1959^2^ and later by Mustard in 1964.^3^ These techniques led to improved survival rates. However, while these procedures restore physiological blood flow, they do not provide anatomical correction, leaving the morphological right ventricle to sustain the systemic circulation. This results in long-term complications such as systemic right ventricle (sRV) heart failure (HF), arrhythmias, tricuspid valve dysfunction, baffle-related dysfunction, and sudden cardiac death (SCD). Subsequent advancements in surgical techniques led to the introduction of the arterial switch operation in 1975^4^, which restored normal circulation and resulted in better outcomes.^5^ Nevertheless, nowadays many patients who underwent an atrial switch procedure continue to require ongoing medical care. Although some large multicenter studies have been published, very long-term longitudinal follow-up has not been reported. In addition, most studies suffer from survival-related selection bias. A comprehensive understanding of long-term effects is crucial for identifying specific risk factors, managing patient expectations and guiding therapeutic decisions, including heart transplantation or cardiac assist device implantation.

The present study is part of a unique cohort of patients with TGA, who underwent Mustard repair at young age and are evaluated every 10 years, with this being the fourth evaluation after the initial surgical correction (Central Illustration). This cohort offers the opportunity to reliably assess mortality and morbidity, but also to investigate changes over time in cardiac function, exercise capacity and subjective quality of life (QoL), and to detect predictors for late outcome.

**Central Illustration:**
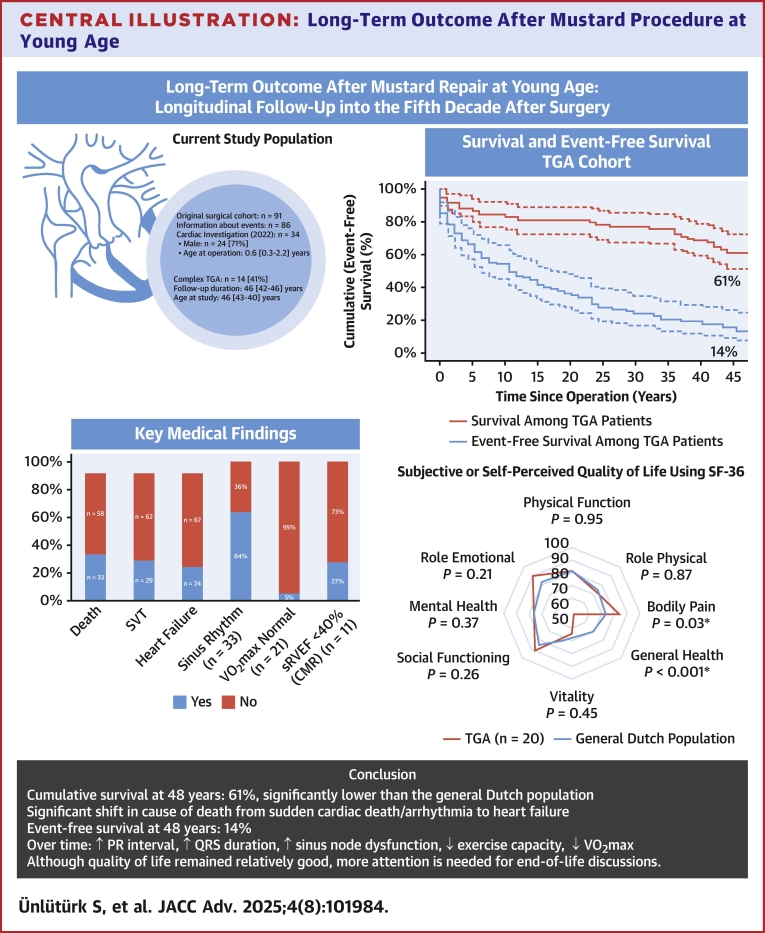
Long-Term Outcome After Mustard Procedure at Young Age This central illustration summarizes long-term outcomes in a cohort of TGA patients who underwent Mustard repair at young age. After a total follow-up of 48 years, cumulative survival was 61% and significantly lower than that of the general Dutch population. Only 14% of patients remained event-free. Over time, a clear shift in cause of death was observed from sudden cardiac death and arrhythmia toward heart failure. Additional findings included progressive sinus node dysfunction, increased PR interval and QRS duration, and declining exercise capacity. Despite these clinical deteriorations, quality of life was relatively preserved. More attention is needed for end-of-life discussionss. CMR = cardiovascular magnetic resonance; SF-36 = 36-Item Short Form Survey; sRVEF = systemic right ventricular ejection fraction; SVT = supraventricular tachycardia; other abbreviations as in Figures 1 and 3.

## Methods

### Study population

This study is a single-center longitudinal cohort study evaluating all consecutive patients with TGA who underwent Mustard repair at the Erasmus Medical Center between 1973 and 1980 at young (<15 years) age. This cohort was previously described in 1990, 2001, and 2012.^6^, ^7^, ^8^ “Complex TGA” was defined as needing closure of a ventricular septal defect and/or correction of pulmonary stenosis (PS) in addition to the Mustard repair. All surviving patients who had participated in at least one of the prior investigations were invited for this new cardiac investigation, which included additional analyses according to the study protocol.

Alongside this extensive cardiological assessment, subjective QoL evaluation was also conducted. In cases where patients were unable to participate in the cardiac investigation, consent was sought to use data available from their medical records, reflecting routine follow-up at our hospital. The study protocol was approved by the local Medical Ethics Committee (MEC 2019-0465) and written informed consent was obtained from all study participants. The study was carried out according to the principles of the Declaration of Helsinki.

### Outcome

Survival status of the entire cohort was obtained from the Dutch National Population Registry and compared to the age-matched general Dutch population (GDP). The primary outcome was overall survival. The secondary outcome was event-free survival, defined as survival without any composite event, including all-cause mortality, cardiac reinterventions (both surgical and interventional), symptomatic arrhythmia, pacemaker/implantable cardioverter-defibrillator (ICD) implantation, stroke, HF, and endocarditis. These adverse events were obtained from the cardiac investigations and medical records. HF was diagnosed when a patient was prescribed HF medication or required hospitalization. Arrhythmias were considered symptomatic if medication was prescribed, hospitalization was necessary, or if patients underwent a cardioversion, pacemaker implantation or ablation procedure (either surgically or catheter-based). Tertiary outcomes included temporal changes of clinical variables, association between cardiac investigation and clinical status, and the outcome of subjective QoL.

### Cardiac investigation

Cardiac investigation included 12-lead surface electrocardiography, 24-hour ambulatory Holter monitoring, cardiopulmonary exercise testing (CPET), transthoracic echocardiography, cardiovascular magnetic resonance (CMR), and laboratory measurements (Supplemental Table 1). Maximal workload was defined as exercise capacity. All echocardiographic studies were conducted and evaluated in compliance with the current guidelines.^9^, ^10^, ^11^, ^12^ However, for the longitudinal design of this study, in some instances old reference values were used. Baffle stenosis was identified by a flow rate exceeding 1.1 m/s within the baffle. Pulmonary hypertension was defined as a mitral regurgitant jet velocity >2.8 m/s in the absence of PS or left ventricular outflow tract obstruction or a pulmonary regurgitant jet velocity >2.2 m/s. The CMR scans from the prior study were re-evaluated following current standards, utilizing the software available today. Both prior CMR data and new findings from this study were analyzed in line with contemporary literature.^8^^,^^13^

Patients’ subjective health was assessed by 36-Item Short Form Survey (SF-36) questionnaire, and the results were compared to the age-matched GDP and with earlier assessments.^14^

### Statistical analysis

Distributions of continuous variables were tested for normality using the Shapiro-Wilk test, and reported as mean ± 1 SD or median (25th-75th percentile) as appropriate. Categorical variables were reported as frequencies and percentages.

To examine differences between independent patient groups, we used the Student’s *t*-test for normally distributed continuous variables, the Mann-Whitney *U* test for non-normally distributed data, and either the chi-square test or Fisher exact test for categorical variables, depending on expected cell counts. We also investigated whether values within individuals changed between visits. To evaluate this, we applied the paired *t*-test for normally distributed continuous variables, the Wilcoxon signed rank test for skewed data, and the McNemar test for paired categorical outcomes.

We then assessed the strength and direction of associations between variables. This was done using Pearson’s correlation for normally distributed continuous variables, Spearman’s rank correlation for nonparametric data, and point-biserial correlation to evaluate relationships between continuous and binary variables.

To analyze repeated measures and track longitudinal changes within individuals across multiple visits, we used linear mixed-effects models for continuous outcomes and generalized linear mixed models for dichotomous outcomes, incorporating random intercepts and slopes to account for presence of multiple measurements per individual. The evaluation moments were included as fixed effects and as random effects. The random effects were modeled with an unstructured covariance matrix. We did not specify an additional within-subject correlation structure. For each outcome, 2 mixed models were constructed: one including both a random intercept and a random slope, and one including only a random intercept. These models were compared using an analysis of variance test, and the model with the best fit was selected for each outcome.

We assessed survival of the study cohort compared to the Dutch reference population. Survival curves for the patient cohort were based on individual mortality dates, while survival curves for the general population were derived from publicly available year-specific mortality probabilities. The Dutch reference population was defined as individuals aged 1 year in 1973, corresponding to the typical age of primary surgical correction in our cohort. Survival at 48 years was then compared using Z-tests. We also stratified survival analysis by transposition complexity. Cumulative incidence curves for nonfatal outcomes were generated using a nonparametric estimator of cumulative incidence functions.

To identify predictors of long-term outcomes such as mortality and HF, we performed univariable Cox proportional hazards regression analyses. The baseline time point was the date of surgery. Variables as assessed at the time of operation and included in the models comprised age at operation, era of operation (pre- or post-1977), intraoperative temperature, aorta clamp time per 5 minutes, pulmonary regurgitation (PR) interval per 10 milliseconds, QRS duration, early postoperative arrhythmias, and simple or complex TGA correction. Variables that showed a statistically significant association with outcomes in the univariable analyses were subsequently included in the multivariable Cox regression model. An additional analysis was conducted using the first structured evaluation in 1990 as the baseline time point, for specific imaging parameters that were measured in 1990, to explore their potential predictive value for mortality and the composite endpoint (mortality and HF).

Finally, to assess the impact of time-varying variables on survival, we used Joint Models, which integrate linear mixed models with Cox regression, allowing for the evaluation of serial measurements in relation to time-to-event outcomes. Time-dependent variables included rhythm on Holter, PR interval, QRS duration, ventricular tachycardia (VT) on Holter, exercise capacity (maximal workload), and subpulmonary left and systemic right ventricular function (spLVF and sRVF) determined via echocardiography.

Statistical analyses were performed using the Statistical Package for Social Sciences (version 28.0, SPSS, Inc, IBM Corp) and R software version 4.3.2 (R Foundation for Statistical Computing). All tests were 2-sided, and *P* values <0.05 were considered statistically significant.

## Results

### Study patients

The original study cohort consisted of 91 consecutive patients (65% male, age at operation 0.7 [0.3-3.2] years). Prior palliation encompassed Blalock-Hanlon atrioseptostomy in 15 (16%) and Rashkind balloon atrioseptostomy in 65 of the 91 patients (71%). During Mustard repair, 36 patients (40%) underwent additional interventions for either ventricular septal defect closure or relief of PS (“Complex Mustard”).

**Figure 1:**
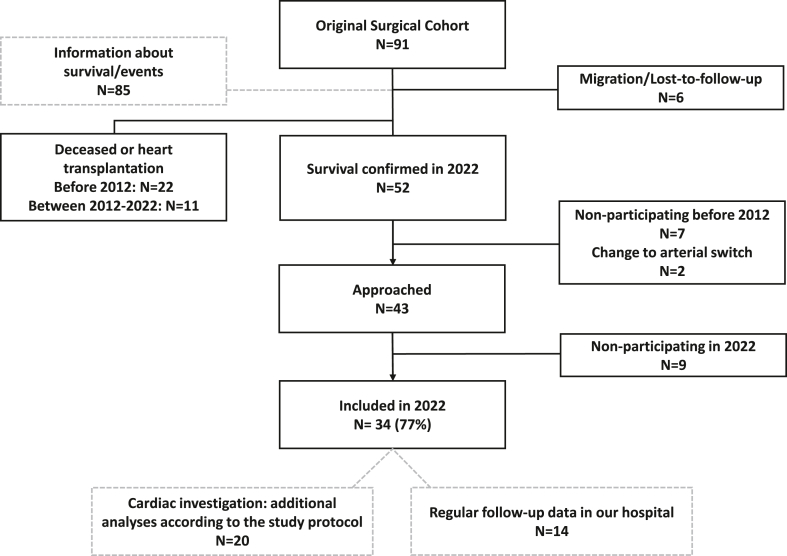
Current Study Participation This flowchart presents the inclusion of TGA patients who previously underwent Mustard repair. From the original surgical cohort of 91 patients, follow-up clinical data were available for 86 patients. A total of 33 patients had died or received a heart transplantation, and 6 patients had either migrated or were lost to follow-up. Two patients were excluded because they underwent an arterial switch operation after the initial Mustard procedure. In 2022, 20 patients participated in comprehensive cardiac investigations, while data from an additional 14 patients were retrieved from routine clinical follow-up at our center. The remaining patients did not participate in the most recent cardiac evaluation. TGA = transposition of the great arteries.

Current patient inclusion is visualized in Figure 1. The original cohort consisted of 91 patients. Thirty-four of the 44 patients (77%) participated in the last evaluation round (2022). Of these, 20 patients provided informed consent for additional cardiac investigations according to the study protocol, while 14 consented to the use of their routine clinical follow-up data from our center. Baseline characteristics are presented in Table 1. Median follow-up was 46 (range: 40-48) years after surgery. There were no differences in baseline characteristics between participating and nonparticipating patients (Supplemental Table 2A).

**Table 1:** Baseline Characteristics

	Total (N = 91)	1990 (n = 58)	2001 (n = 54)	2012 (n = 50)	2022 (n = 34)
Male	59 (65%)	40 (69%)	38 (70%)	32 (64%)	24 (71%)
Age at operation, y	0.7 [0.3-3.2]	0.7 [0.3-2.5]	0.7 [0.3-2.5]	0.7 [0.4-2.5]	0.6 [0.3-2.2]
Prior palliation	83 (91%)	55 (95%)	52 (96%)	47 (94%)	33 (97%)
Left atrium saturation, %	97 [89-99]	97 [86-99]	97 [88-98]	96 [86-98]	98 [86-99]
Aortic clamp time, min	58 [50-69]	59 [50-69]	59 [51-68]	58 [50-69]	56 [50-62]
Hypothermia					
Temperature <20 °C	60 (66%)	44 (76%)	40 (74%)	37 (74%)	27 (79%)
Temperature 20-35 °C	22 (24%)	11 (19%)	11 (20%)	10 (20%)	7 (21%)
Temperature unknown	9 (10%)	3 (5%)	3 (6%)	3 (6%)	0 (0%)
Complex TGA	36 (40%)	21 (36%)	18 (33%)	18 (36%)	14 (41%)
Postoperative arrhythmias	9 (10%)	9 (16%)	8 (15%)	7 (14%)	3 (9%)
Pacemaker before 1990	12 (13%)	8 (14%)	8 (15%)	6 (12%)	5 (15%)
Follow-up, y	-	13 [12-16]	25 [24-28]	35 [34-38]	46 [42-46]
Age at study, y	-	14 [13-18]	26 [25-30]	36 [34-40]	46 [43-49]

TGA = transposition of the great arteries.

### Survival

**Figure 2:**
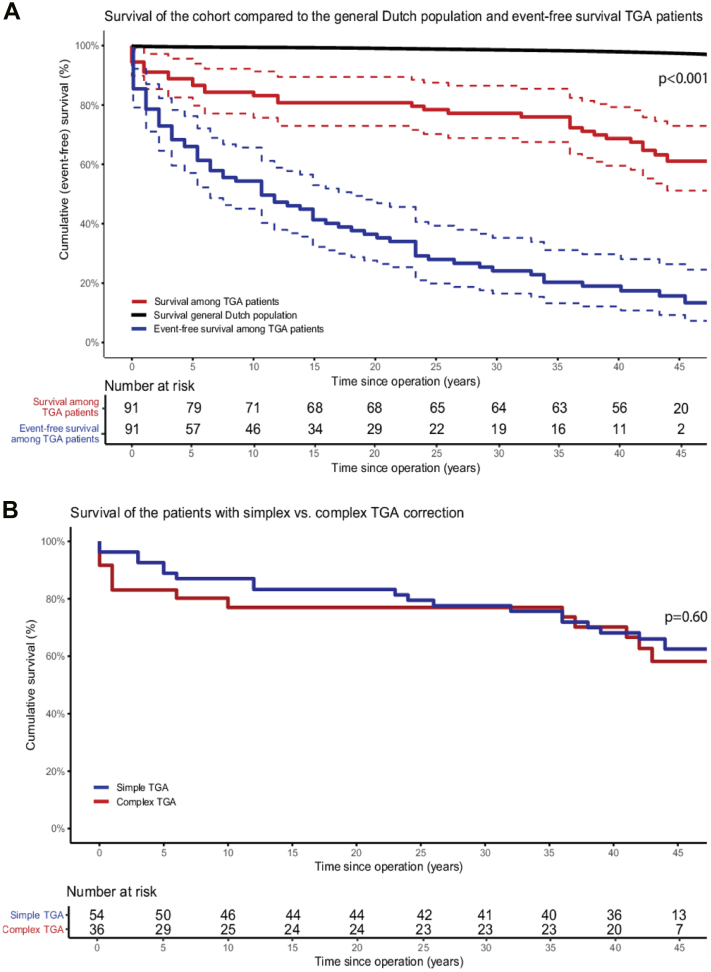
Survival and Event-Free Survival of the TGA Cohort (A) Survival curve of the TGA cohort (red) compared to the Dutch reference population (black), and event-free survival (blue) (B) Survival of the patients with simple (blue) vs complex TGA correction (red). A shows the cumulative survival (red) and event-free survival (blue) of the TGA cohort, compared to age- and sex-matched survival of the general Dutch population (black). After nearly 5 decades of follow-up, cumulative survival was 61%, significantly lower than the general population (*P* < 0.001), and event-free survival was only 14%. B compares survival between patients with simple TGA (blue) and complex TGA (red). No statistically significant difference in long-term survival was observed between the 2 groups (*P* = 0.60). Abbreviation as in Figure 1.

Survival status was obtained in 85 of 91 patients (93%). Six patients moved abroad or were untraceable. Cumulative survival after surgical correction was 84% at 10 years, 77% at 30 years, and 61% (51% to 73%) at 48 years (Figure 2A). Survival was significantly lower compared to the GDP (*P* < 0.001). The cumulative survival of patients with simple vs complex TGA was not statistically significantly different (Figure 2B) (63% vs 58%, *P* = 0.60). In total, 33 of the 91 patients died or received heart transplantation, 6 died within 30 days after surgery.

Mortality rate was 1.1 per 100 patient-years (33 deaths or heart transplantation/3001 patient-years, overall). In the last 10 years, 9 patients died, of which 4 due to end-stage HF. In Supplemental Table 3, an overview of all causes of death is provided.

### Major cardiovascular events

Event-free survival at 48 years was 14% (Figure 2A). The event-free survival was 15% for simple TGA correction and 12% for complex TGA patients (*P* = 0.60). An overview of all recorded events is provided in Supplemental Table 4.

The cumulative incidence of HF was 37%. Cardiac medication use is reported in Supplemental Table 2B. The cumulative incidence of symptomatic arrhythmias was 39%. In the last decade, 8 patients developed new symptomatic arrhythmias. Eight were supraventricular, including 2 atrial flutter and 2 atrial fibrillation and 4 were ventricular (2 had both). Two patients had atrial tachycardia of which one developed tachy-brady syndrome and underwent pacemaker implantation. One patient developed a sick-sinus syndrome and another a total atrioventricular block, with both needing a pacemaker implantation. A history of supraventricular tachycardia (SVT) was negatively correlated with VO_2_max (r_pb_ = −0.44, *P* = 0.04) and positively correlated with both VT (*P* = 0.02) and HF (*P* < 0.001) (Supplemental Table 5). Among patients with a pacemaker, 56% developed HF, compared to 14% in those without (*P* < 0.001). In total, 27 patients needed a pacemaker implantation (19 endocardial, 8 epicardial). When comparing the patients with an endocardial vs epicardial pacemaker, SVT occurred in 84% vs 50% (median time to event: 32 vs 23 years, *P* = 0.15), VT in 26% vs 25% (median time to event: 42 vs 25 years, *P* = 1.00), and HF in 63% vs 38%, respectively (median time to event: 34 vs 28 years, *P* = 0.40).

The cumulative incidence of reintervention at 48 years was 39%. In the last decade, 3 patients required ≥1 reinterventions: tricuspid valve replacement (TVR) and baffle revision (n = 1), TVR (n = 1), tricuspid valve plasty and baffle revision (n = 1). In both cases, baffle revision was not the primary indication for surgery. In total, 24 patients underwent baffle revision and 6 patients underwent tricuspid valve annuloplasty or replacement.

In total 4 patients suffered a stroke, all in the last decade, and all had complex TGA. Two had a baffle leak, a history of SVT and a pacemaker. One patient had a history of SVT and VT. In total, 1 patient was diagnosed with endocarditis. This patient also had a pacemaker.

### Cardiac investigation

**Figure 3:**
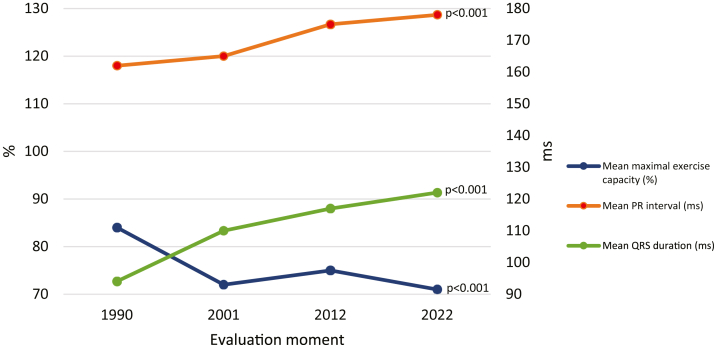
Trend Over Time Diagnostic Measurements This figure displays temporal trends in key diagnostic parameters among patients who underwent repeated cardiac investigations over the years. Findings demonstrate a progressive increase in PR interval and QRS duration, reflecting electrical conduction abnormalities. Additionally, exercise capacity declined over time, suggesting functional clinical deterioration.

Electrocardiography, Holter, CPET, and echocardiography findings are summarized in Table 2, Figure 3, and Supplemental Figure 1. Sinus rhythm was present in 64%. There was a significant increase in PR interval and QRS duration over the years (both *P* < 0.001), with now 21% having a PR interval >200 ms and 30% having a QRS duration >120 ms. CPET results showed a reduced exercise capacity in 77% of patients and 95% had a reduced VO_2_max. QRS duration was positively correlated with HF (r_pb_ = 0.58, *P* = 0.001), while exercise capacity (r_pb_ = −0.47, *P* = 0.03) and VO_2_max (r_pb_ = −0.52, *P* = 0.02) were negatively correlated with HF.

**Table 2:** Diagnostic Measurements

	1990 (n = 58)	2001 (n = 54)	2012 (n = 50)	2022 (n = 34)	*P* for Trend^a^
Electrocardiography	n = 58 (100%)	n = 54 (100%)	n = 47 (94%)	n = 33 (97%)	
Rhythm					0.38^b^
Sinus	40 (69%)	34 (63%)	31 (66%)	21 (64%)	
Atrial	6 (10%)	5 (9%)	5 (11%)	2 (6%)	
Atrial fibrillation/flutter	1 (2%)	0 (0%)	3 (6%)	0 (0%)	
Nodal	7 (12%)	7 (13%)	1 (2%)	0 (0%)	
Pacemaker	4 (7%)	8 (15%)	7 (15%)	9 (27%)	
Other	0 (0%)	0 (0%)	0 (0%)	1 (3%)	
Heart rate, beats/min	73 ± 16	71 ± 16	65 ± 12	69 ± 12	0.02
PR interval, ms	162 ± 42	165 ± 23	175 ± 43	178 ± 30	<0.001
PR interval >200 ms	1 (2%)	1 (3%)	7 (21%)	5 (21%)	<0.001
QRS duration, ms	94 ± 11	110 ± 17	117 ± 19	122 ± 19	<0.001
QRS duration >120 ms	0 (0%)	11 (25%)	13 (33%)	8 (30%)	0.002
24-hour Holter	n = 57 (98%)	n = 50 (93%)	n = 37 (74%)	n = 20 (59%)	
Mean heart rate, beats/min			73 ± 14	72 ± 8	0.45
Sinus node dysfunction^c^	18 (32%)	30 (60%)	19 (51%)	13 (65%)	0.008
Paroxysmal atrial fibrillation/flutter	1 (2%)	0 (0%)	4 (9%)	0 (0%)	NA
VT 3-10 complexes	3 (5%)	4 (8%)	6 (16%)	4 (19%)	0.59
VT >10 complexes	0 (0%)	0 (0%)	0 (0%)	0 (0%)	NA
CPET	n = 49 (84%)	n = 49 (91%)	n = 36 (72%)	n = 21 (64%)	
Maximal heart rate, %	86 [80-90]	87 [79-92]	85 [76-92]	81 [74-92]	0.03
Maximal exercise capacity, %	84 [74-93]	74 [64-84]	73 [68-87]	72 [64-83]	<0.001
Maximal exercise capacity <85%	24 (49%)	38 (78%)	25 (69%)	17 (77%)	0.005
VO_2_ max, %^d^			69 [54-80]	61 [47-75]	0.07
VO_2_ max <85%			20 (83%)	20 (95%)	1.0
RER max			1.3 [1.2-1.4]	1.3 [1.2-1.4]	0.13
Arrhythmia	5 (10%)	5 (10%)	6 (17%)	5 (23%)	0.12
Echocardiographic parameters^e^	n = 58 (100%)	n = 53 (98%)	n = 47 (94%)	n = 32 (94%)	
Normal systolic sRV function	40 (69%)	3 (6%)	1 (2%)	0 (0%)	<0.001
Normal systolic spLV function	57 (98%)	50 (94%)	30 (64%)	24 (75%)	0.001
Valve regurgitation (>trace)					
AR	4 (7%)	5 (9%)	7 (15%)	5 (31%)	0.003
MR	8 (14%)	12 (23%)	11 (23%)	5 (31%)	0.02
PR	15 (26%)	27 (51%)	27 (57%)	8 (29%)	0.16
TR	36 (62%)	45 (85%)	43 (92%)	25 (78%)	0.03
Severe tricuspid regurgitation	1 (2%)	10 (19%)	17 (38%)	5 (17%)	0.80
Vmax MR, m/s	2.9 [2.6-3.9]	2.5 [2.0-4.1]	2.8 [2.2-3.8]	2.4 [2.2-2.9]	0.49
Vmax PR, m/s	1.9 [1.7-2.1]	1.9 [1.6-2.3]	2.0 [1.4-2.5]	1.9 [1.6-2.3]	0.51
Baffle obstruction		6 (11%)	10 (21%)	6 (18%)	0.32
Baffle leakage		1 (2%)	2 (4%)	2 (6%)	0.22

AR = aortic regurgitation; CPET = cardiopulmonary exercise testing; MR = mitral regurgitation; PR = pulmonary regurgitation; RER max = Respiratory Exchange Ratio maximum, a measure of the ratio between carbon dioxide production and oxygen consumption at peak exercise; spLV = subpulmonary left ventricular; sRV = systemic right ventricular; TR = tricuspid regurgitation; Vmax = maximal velocity found with Doppler echocardiography; VO_2_ max = maximum rate of oxygen consumption attainable during physical exertion; VT = ventricular tachycardia.

a Obtained from (generalized) linear mixed models.

b Sinus rhythm vs other rhythms.

c Surface ECG criteria for sinus node dysfunction according to the Kugler criteria.

d No VO_2_max available in 2 patients.

e N varies between 3 and 31.

CMR analysis was performed in 11 patients (Table 3). The median sRV ejection fraction (EF) was 35% (32%-40%). Seventy-three percent of the patients had a sRVEF <40%. The systemic right ventricular end-diastolic and end-systolic volume (RVEDV and sRVESV) index and sRVEF were 129 (112-147), 86 (73-107) mL/m^2^, and 33% (32%-35%), respectively, in the patients with a diminished exercise capacity and 104 (92-119), 60 (53-71) mL/m^2^, and 43% (38%-47%), respectively, in the patients with normal exercise capacity (*P* = 0.10, 0.03, and 0.02, respectively).

**Table 3:** Cardiac Magnetic Resonance Imaging Analysis

	2012		2012 Reanalysis^a^			2022			*P* Value^b^
	Median	25th-75th percentile	n (%)	Median	25th-75th percentile	n (%)^c^	Median	25th-75th percentile	
Systemic right ventricle									
EDVi (mL/m^2^)	93	[70-107]	24 (48%)	115	[108-138]	11 (32%)	119	[97-131]	0.14
ESVi (mL/m^2^)	41	[35-56]	24 (48%)	70	[65-90]	11 (32%)	76	[56-90]	0.04
EF (%)	47	[40-60]	24 (48%)	38	[36-41]	11 (32%)	35	[32-40]	0.14
EF<40%				15 (63%)			8 (73%)		
Subpulmonary left ventricle									
EDVi (mL/m^2^)	60	[55-74]	24 (48%)	65	[63-75]	11 (32%)	67	[58-75]	0.11
ESVi (mL/m^2^)	21	[17-30]	24 (48%)	27	[23-33]	11 (32%)	28	[25-34]	0.31
EF (%)	63	[55-68]	24 (48%)	61	[52-63]	11 (32%)	55	[53-62]	0.68
EF<40%				0 (0%)			0 (0%)		

EDVi = end-diastolic volume index; EF = ejection fraction; ESVi = end-systolic volume index.

a Reanalysis performed by the same operator according to the current guidelines.

b 2012 reanalysis vs2022.

c 23 patients did not undergo CMR due to having a pacemaker, claustrophobia, or refusal to participate.

Laboratory measurements are reported in Supplemental Table 6. Median N-terminal pro–B-type natriuretic peptide (NT-proBNP) level was 45.0 pmol/L (30.0–63.0 pmol/L) and was elevated in 97% of the patients. NT-proBNP was negatively correlated with exercise capacity (R = −0.66, *P* < 0.001), VO_2_max (R = −0.74, *P* < 0.001), and sRVEF (R = −0.86, *P* < 0.001). There was a positive correlation with systemic RVEDV (R = 0.66, *P* = 0.03) and RVESV (R = 0.74, *P* = 0.01).

### Predictors of clinical outcome

Results of the Cox regression analyses that included operative characteristics as baseline variables are presented in Table 4. Early postoperative arrhythmias were associated with an increased risk of late symptomatic arrhythmias (*P* = 0.03), while prolonged QRS duration was associated with HF (*P* = 0.009). The additional analysis wherein we used specific parameters from 1990 as baseline variables showed that sRVF was associated with both mortality and with the composite endpoint of mortality and HF (Supplemental Table 7). Joint models showed a significant association between serially measured spLVF and mortality (HR: 2.21; 95% CI: 1.01-6.95; *P* = 0.04).

**Table 4:** Predictors of All-Cause Mortality, Reintervention, Arrhythmias, and Heart Failure

Endpoint^a^	Univariable Model			Multivariable Model		
	HR	95% CI	*P* Value	HR	95% CI	*P* Value
All-cause mortality (n = 33)						
Age at operation	1.23	[1.08-1.41]	**0.002**	1.03	[0.85-1.25]	0.77
Operated after 1977	0.27	[0.12-0.63]	**0.002**	0.44	[0.16-1.19]	0.11
Hypothermia during surgery	0.27	[0.13-0.55]	**<0.001**	0.44	[0.17-1.12]	0.09
Aortic clamp time (per 5 min)	1.06	[0.93-1.20]	0.41			
PR interval after surgery (per 10 milliseconds)	1.09	[0.88-1.36]	0.44			
QRS duration after surgery^b^	1.11	[0.43-2.88]	0.83			
Early postoperative arrhythmias	2.13	[0.81-5.57]	0.12			
Complex or simple TGA	0.90	[0.44-1.82]	0.76			
Reintervention (n = 34)^c^						
Age at operation	0.94	[0.76-1.16]	0.54			
Operated after 1977	1.78	[0.80-3.92]	0.16			
Hypothermia during surgery	1.16	[0.46-2.94]	0.76			
Aortic clamp time (per 5 min)	1.002	[0.88-1.15]	0.97			
PR interval after surgery (per 10 milliseconds)	1.03	[0.90-1.17]	0.69			
QRS duration after surgery^b^	0.37	[0.09-1.57]	0.18			
Early postoperative arrhythmias	0.57	[0.08-4.25]	0.58			
Complex or simple TGA	1.29	[0.55-3.02]	0.56			
Late symptomatic arrhythmias (n = 33)						
Age at operation	1.47	[1.24-1.73]	**<0.001**	1.24	[0.99-1.56]	0.06
Operated after 1977	0.35	[0.17-0.74]	**0.006**	0.77	[0.30-2.01]	0.60
Hypothermia during surgery	0.22	[0.11-0.47]	**<0.001**	0.45	[0.15-1.17]	0.10
Aortic clamp time (per 5 min)	0.92	[0.80-1.05]	0.23			
PR interval after surgery (per 10 milliseconds)^d^	1.22	[1.05-1.41]	**0.009**			
QRS duration after surgery^b^	2.29	[1.01-5.21]	**0.047**	1.82	[0.77-4.28]	0.17
Early postoperative arrhythmias	3.11	[1.04-9.28]	**0.04**	3.59	[1.12-11.49]	**0.03**
Complex or simple TGA	0.79	[0.39-1.61]	0.52			
Heart failure (n = 24)						
Age at operation	1.18	[0.99-1.41]	0.07			
Operated after 1977	0.90	[0.37-2.16]	0.81			
Hypothermia during surgery	0.69	[0.29-1.65]	0.41			
Aortic clamp time (per 5 min)	1.06	[0.92-1.23]	0.43			
PR interval after surgery (per 10 milliseconds)	1.05	[0.90-1.24]	0.56			
QRS duration after surgery^b^	3.23	[1.34-7.74]	**0.009**	3.23	[1.34-7.74]	**0.009**
Early postoperative arrhythmias	1.82	[0.53-6.24]	0.34			
Complex or simple TGA	0.76	[0.34-1.72]	0.51			
Composite endpoint (mortality and heart failure) (n = 57)						
Age at operation	1.16	[1.03-1.31]	**0.02**	1.08	[0.91-1.27]	0.40
Operated after 1977	0.62	[0.34-1.15]	0.13			
Hypothermia during surgery	0.47	[0.27-0.84]	**0.01**	0.59	[0.27-1.26]	0.17
Aortic clamp time (per 5 min)	1.05	[0.95-1.17]	0.33			
PR interval after surgery (per 10 milliseconds)	0.98	[0.83-1.15]	0.78			
QRS duration after surgery^b^	1.42	[0.68-2.94]	0.35			
Early postoperative arrhythmias	1.60	[0.68-3.80]	0.29			
Complex or simple TGA	0.87	[0.50-1.53]	0.63			

Abbreviation as in Table 1.

a Cox regression, adjusted for sex.

b The variable “QRS duration after surgery’’ is dichotomous with 0 representing normal and 1 representing prolonged (>120 ms). Absolute numbers are not available.

c Baffle interventions are classified as reintervention.

d The variable ‘‘PR interval after surgery’’ is not included in the multivariable model due to a significant amount of missing variables (n = 47/91).

### Quality of life

**Figure 4:**
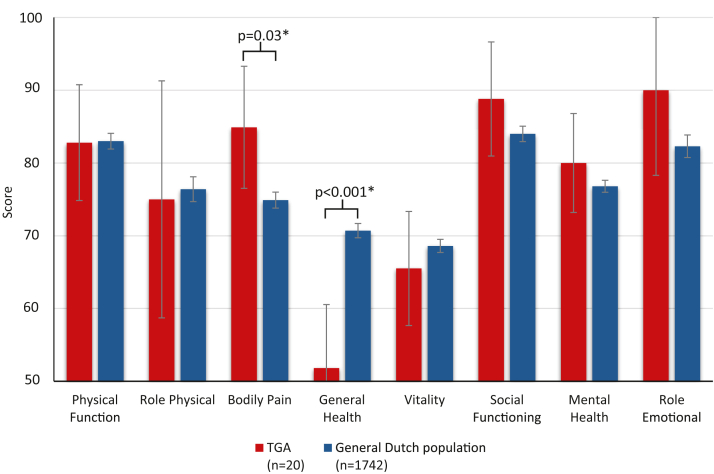
36-Item Short Form Survey Results of the TGA Cohort This figure compares 36-Item Short Form Survey health survey results from the TGA cohort with normative data from the general Dutch population. While physical functioning, vitality, and mental health domains were relatively preserved, TGA patients scored significantly lower in general health and bodily pain domains (*P* < 0.001 and *P* = 0.03, respectively). These results highlight a relatively stable self-perceived quality of life, despite considerable cardiac morbidity in this long-term follow-up population. Abbreviation as in Figure 1.

Subjective QoL using the SF-36 survey for patients and the age-matched GDP are shown in Figure 4, Supplemental Figures 2 and 3, and Supplemental Table 8. Patients scored significantly better on domain bodily pain (*P* = 0.03) and worse on general health (*P* < 0.001) compared to the GDP. Over time, patients showed a less favorable subjective QoL. There was no significant difference between male and female patients. Patients with SVT scored less for the domain general health (41.5 vs 51.8, *P* = 0.02). NT-proBNP was negatively correlated with the domains physical function (R = −0.69, *P* = 0.002), vitality (R = −0.64, *P* = 0.005), and social functioning (R = −0.54, *P* = 0.02).

## Discussion

In this study, we describe the survival and clinical outcomes of an unbiased cohort of patients with TGA who underwent Mustard repair at young age and are now in their fifth decade of follow-up. Regular assessments every decade have allowed tracking changes in clinical outcomes and cardiac function over time, as well as to identify predictors of late outcomes.

### Mortality

Cumulative survival at 48 years was 61% (51%-73%), and significantly lower than the GDP. Moreover based on the survival curve, ongoing mortality is to be expected in the coming years. Consistent with existing literature, arrhythmias and especially HF were the leading cause of mortality.^15^ We observed an evident shift from SCD, most likely due to arrhythmia, in the earlier period to end-stage HF in the last 15 years. To our knowledge, there are no studies with a similarly long follow-up period. Several studies reported survival rates of 60 to 89% at 30 to 35 years.^4^^,^^16^, ^17^, ^18^ Venkatesh and colleagues conducted a meta-analysis showing an average survival rate of 65% at 40 years.^19^ In our cohort, not only mortality but also morbidity was significant with an event-free survival rate of only 14% at 48 years.

These findings highlight the need for health care systems to prepare for the specific challenges faced by this population.

### Heart failure and ventricular function

Over the 48-year follow-up period, the cumulative incidence of HF was 37% with 6 new HF cases in the last decade. Most studies on medical therapies for failing sRV show limited benefits of traditional HF medication: a meta-analysis found no significant effects of angiotensin-converting enzyme inhibitors, angiotensin receptor neprilysin inhibitor, beta-blockers, and mineralcorticosteroids receptor antagonists on CMR dimensions and functions, or CPET results, nor did they reduce HF risk or mortality.^20^ Early results from the DAPA-SERVE trial show promising results of dapagliflozin (a sodium-glucose transport protein 2 inhibitor) and large prospective studies are eagerly awaited.^21^ While sacubitril/valsartan has not demonstrated an enhancement in long-term survival, it has been associated with a reduction in adverse effects^22^ and significant improvements in NT-proBNP levels (a biomarker of cardiac wall stress), physical activity, QoL, and sRVF.^23^ With the shift in cause of death toward HF, it may be beneficial to redefine HF beyond the traditional symptom-based approach, incorporating NT-proBNP levels, NYHA functional class, and VO_2_max.^24^^,^^25^ Even though in our study the majority of patients were classified as NYHA functional class I and thus were considered asymptomatic, this will not reflect the severity of their condition. Patients may downplay their symptoms, which can cause discrepancies between their self-reported NYHA functional class and objective exercise metrics, and hence the true severity of the underlying cardiac condition. Therefore, integrating specific criteria into treatment protocols may improve patient management by facilitating earlier and more targeted interventions, including not only medication but also considering ICD implantation, right-ventricular assist device or even heart transplantation. NT-proBNP and VO_2_max trends, alongside clinical assessments could be used to initiate the discussion, changing from previously waiting for clinically evident HF, to proactively initiate treatment promptly at the first signs of deterioration as is a common practice in our institution nowadays.^15^

### Arrhythmias and sudden cardiac death

The cumulative incidence of symptomatic arrhythmias was 39%, mostly SVT. Risk factors for SCD in patients with sRV include atrial arrhythmias^26^, while ventricular arrhythmias were found to be related to systemic ventricular dysfunction.^27^ Preventive strategies should combine medical and lifestyle interventions, as growing evidence supports that an active lifestyle is beneficial, also for congenital heart disease (CHD) patients.^28^ While negative inotropic antiarrhythmic drugs can help control arrhythmias and reduce oxygen demand, they may decrease cardiac output and adversely affect ventricular function. In selected patients, ICD implantation can be lifesaving and is warranted. However, anatomical issues can make this challenging and lead implantation may cause obstruction in relatively narrow superior systemic venous baffles.^29^ Patients receiving an ICD face the stress of appropriate but also inappropriate shocks and have an elevated endocarditis risk.^30^^,^^31^ Ladouceur et al developed a risk prediction model to guide the consideration of primary prevention ICD implantation.^32^

In our cohort, 21 of the evaluated 33 patients (64%) maintained sinus rhythm, a slight decrease over time. Notably, sinus node dysfunction has significantly increased over time, from 32% in 1990 to 65% in 2022. The absence of sinus rhythm is frequently observed in patients following the Mustard repair and is linked to poorer cardiac outcomes.^15^^,^^33^ In our cohort, pacemaker implantation was necessary in 27 patients of whom 8 received an epicardial pacemaker.

Permanent pacing often requires repeat interventions, particularly with epicardial pacing, which has a higher rate of lead failures and significantly lower durability.^34^ Moreover, chronic pacing can potentially exacerbate ventricular dilation and failure, as well as tricuspid regurgitation. In our cohort, the pacemaker group had a lower average VO_2_max and a higher prevalence of HF. We cannot conclude that this is the effect of pacing or rather that pacemaker implantation was needed in sicker patients, but further attention is clearly needed.

### Reintervention

The cumulative incidence of reintervention was 39%. However, in the last decade, only 3 patients required a reintervention which was significantly less compared to earlier follow-up rounds. Issues related to baffle problems appear to have been effectively addressed in previous decades. Over the past decade, 3 patients underwent tricuspid valve interventions; at baseline, 2 of these patients had poor sRVF and one had moderate sRVF. Although these limited numbers prelude firm conclusions, echocardiographic assessment following the interventions showed no improvement in sRVF. The goal of tricuspid valve intervention is to preserve tricuspid function and stabilize sRVF and hopefully improve patients' clinical condition. The optimal timing for tricuspid valve plasty or TVR in the management of tricuspid regurgitation remains a topic of debate. Given the lack of improvement in sRVF and the increased risk of complications associated with TVR, along with inconsistent evidence on survival benefits, the necessity and timing of tricuspid valve intervention require further evaluation and individualized consideration. For now, we cannot demonstrate an added value.

### Other complications

In our cohort, 4 patients experienced a stroke; 2 of them had baffle leakage and one a baffle stenosis. As flow velocity in the baffles is low, there is an increased risk of thrombus formation. This raises the question of whether proactive measures, such as routine anticoagulation therapy, should be implemented. Importantly, 3 patients had a pacemaker (2 endocardial, 1 epicardial). While a low threshold for initiating anticoagulation in patients with concurrent SVT, starting prophylactic anticoagulation in all Mustard patients might be an excessive approach. However, anticoagulation may be considered in patients with moderate CHD and a pacemaker, as this is a risk factor for thromboembolic events.^35^ In our cohort, only one case of endocarditis occurred. Moore et al found endocarditis to be more common in CHD in general, although the incidence of endocarditis in TGA patients was also low.^36^

### Predictors of clinical outcome

Our results identify early postoperative arrhythmias as a predictor of late symptomatic arrhythmias, confirming previous findings. Moreover, prolonged QRS duration was found to be a predictor of HF. Additional analyses revealed that a reduced sRVF in 1990 predicted both mortality and the composite endpoint (mortality and HF). Furthermore, in the Joint Models, decline in serially measured spLVF was significantly associated with mortality.

Although long-term mortality risk was associated with severe tricuspid valve regurgitation, NYHA functional class III or higher, HF hospitalization, complex TGA, and Mustard repair by others^37^, we were unable to replicate these findings, probably due to the limited sample size.

### Subjective quality of life

At the last follow-up, patients scored well on self-perceived QoL and even significantly better on the domain bodily pain compared to the GDP. However, patients with TGA reported worse scores in general health compared to the GDP and compared previous assessment. This was accompanied by increasing major cardiac events and reduced VO_2_max. Conflicting literature exists regarding the QoL of CHD patients. Lane et al found a decreased QoL in patients after surgical correction, while Moons et al findings were more positive.^38^^,^^39^ Discrepancies in QoL outcomes may be influenced by varying perceptions of the condition across different regions, particularly between Europe and the United States, where access to high-quality health care and insurability might play a role.

### Advanced therapies for patients in later stages

In a select subgroup of patients in later stages, advanced treatment options may be considered. Heart transplantation and mechanical assist devices may be considered, though anatomical barriers and donor shortages remain major limitations. In our study, 2 patients underwent heart transplantation, highlighting its rarity yet potential value in end-stage HF.^40^ Right ventricular assist device use remains infrequent due to variable outcomes, and careful timing of such interventions is crucial. In our cohort, catheter ablation was performed in 8 patients, reflecting a broader trend despite limited long-term data. Wu et al reported high acute success for SVT ablation post-atrial switch, though intra-atrial reentrant tachycardia recurrence is ∼30%, sometimes requiring redo procedures.^41^

Resynchronization therapy offers benefits in patients with ventricular dysfunction and conduction delay, improving ventricular function and possibly reducing pacemaker-related complications when tailored to anatomical specifics.^42^

As the outlook for these patients is not very optimistic, timely discussions on end-of-life care are also warranted. Patients often perceive their life expectancy as normal, while cardiologists are more pessimistic.^43^ In this study, only 14.9% of patients have discussed end-of-life care, while 49.3% expressed a desire to do so. Effective advanced care planning is essential, and barriers for health care professionals to take the initiative to start these important conversations, should be tackled.^44^

### Study limitations

Due to the limited number of patients in this study, findings should be interpreted with caution. Nonetheless, this cohort provides, to our knowledge, the longest follow-up period available and constitutes a longitudinally studied, unbiased group of Mustard patients.

Survival data were accessible for 91% of the original cohort, while 77% of eligible survivors were included in the present study. Notably, there were no discernible differences between participating and nonparticipating patients, thus mitigating potential selection bias.

Nevertheless, the sample size is smaller compared to previous evaluation moments, which makes it more difficult to achieve sufficient statistical power to draw firm conclusions.

Furthermore, diagnostic techniques have undergone significant advancements in recent decades. Consequently, in certain instances, we were compelled to utilize techniques consistent with those employed in the past to conduct our longitudinal analysis and elucidate temporal changes. In addition, the new techniques that emerged during the follow-up period of this study were also integrated into our analysis. Lastly, the 1990 echocardiography data were not complete.

## Conclusions

Survival after Mustard repair for TGA was 61% (51% to 73%) at 48 years. Over the past decade, there was a shift in cause of death from SCD/arrhythmia to HF. Event-free survival was 14% at 48 years, warranting optimization of care for especially HF and arrhythmias with special attention dedicated to pacemaker implantation. Although QoL remained relatively good, given the high burden of morbidity, more attention is needed for end-of-life discussions.

Perspectives **COMPETENCY IN MEDICAL KNOWLEDGE 1:** This study provides long-term outcome data up to 48 years following Mustard repair in TGA patients. Findings show a shift in mortality from SCD to HF, with progressive sRV dysfunction, arrhythmias, and declining exercise capacity over time.

**COMPETENCY IN MEDICAL KNOWLEDGE 2:** Despite substantial morbidity and a low event-free survival rate of 14%, patient-reported QoL remained relatively stable. These results highlight the complex, long-term clinical course of this patient group. Over time, timely consideration of advanced therapies and end-of-life discussions may become increasingly relevant.

**TRANSLATIONAL OUTLOOK:** Future research should focus on identifying predictors of adverse outcomes and exploring interventions aimed at preserving sRVF, advanced treatment options and reducing arrhythmic burden in this unique population.

## Funding support and author disclosures

This work was supported by Stichting “De Hoop Leven” and “Thoraxfoundation.” The authors have reported that they have no relationships relevant to the contents of this paper to disclose.
