# Supplementary material for: Long-Term Outcome After Mustard Repair at Young Age: Longitudinal Follow-Up Into the Fifth Decade After Surgery
Source: JACC Adv. 2025 Jul 11;4(8):101984. doi: 10.1016/j.jacadv.2025.101984 (PMC12275045; doi:10.1016/j.jacadv.2025.101984)
Supplement: Supplemental Material [file mmc1.pdf]

Supplementary Table 1. Vendor and reference values of the used laboratory measurements.

Laboratory measurement	Vendor	Reference value
Creatinin	Cobas 8000, Roche diagnostics, Basel Switzerland.	male 65-115 umol/L, female 55-90 umol/L)
NT-proBNP	Cobas 8000, Roche diagnostics, Basel Switzerland.	< 15 pmol/L)
HS-Troponin T	Cobas 8000, Roche diagnostics, Basel Switzerland.	<14 ng/L
HS-CRP	Cobas 8000, Roche diagnostics, Basel Switzerland.	<10 mg/L
Total cholesterol	Cobas 8000, Roche diagnostics, Basel Switzerland.	2.9-6.5 mmol/L
LDL cholesterol	Cobas 8000, Roche diagnostics, Basel Switzerland.	2.59-4.12 mmol/L
HDL cholesterol	Cobas 8000, Roche diagnostics, Basel Switzerland.	>1.55 mmol/L
Triglycerids	Cobas 8000, Roche diagnostics, Basel Switzerland.	<2.0 mmol/L
HbA1c	Menarine diagnostics, Florence Italy	26-42 mmol/mol Hb
TSH	Lumipulse, Fujirebio diagnostics, Malvern USA	0.56-4.27 mU/L
FT4	Lumipulse, Fujirebio diagnostics, Malvern USA	13.5-24.3 pmol/L
Hb	XN 9000, Sysmex diagnostics, Hamburg Germany	male 8.6-10.5 mmol/L, female 7.5-9.5 mmol/L

CRP= C-reactive protein; FT4= free thyroxine; Hb= haemoglobin; HbA1C= Haemoglobin A1C; HDL= high-density lipoprotein; LDL= low-density lipoprotein; NT-proBNP= N-terminal prohormone of brain natriuretic peptide; TSH= thyroid stimulating hormone

Supplementary Table 2A. Participants vs. non-participants

	**Participation 2022 N=34**	**No participation 2022*** **N=57**	**P- value**
Male	24 (71%)	35 (61%)	0.38
Age at operation, years	0.6 [0.3-2.2]	0.8 [0.3-3.5]	0.70
Prior palliation	33 (97%)	50 (88%)	0.13
Left atrium saturation, %	98 [86-99]	96 [90-98]	0.46
Aortic clamp time, minutes	56 [50-62]	61 [50-70]	0.19
Hypothermia			0.14
Temperature <20°C	27 (79%)	35 (62%)	
Temperature 20-35°C	7 (21%)	19 (33%)	
Temperature unknown	0 (0%)	3 (5%)	
Complex TGA	14 (41%)	22 (39%)	0.81
Postoperative arrhythmias	3 (9%)	6 (11%)	0.79
Pacemaker before 1990	5 (15%)	7 (12%)	0.28
Follow-up, years	46 [42-46]		
Age at study, years	46 [43-49]		

*Deceased and emigrated patients

	**Participation 2022 N=34**	**No participation 2022*** **N=24**	**P- value**
Male	24 (71%)	14 (58%)	0.33
Age at operation, years	0.6 [0.3-2.2]	0.6 [0.3-1.1]	0.33
Prior palliation	33 (97%)	19 (79%)	**0.03**
Left atrium saturation, %	98 [86-99]	97 [88-99]	0.70
Aortic clamp time, minutes	56 [50-62]	60 [50-70]	0.19
Hypothermia			0.42
Temperature <20°C	27 (79%)	21 (88%)	
Temperature 20-35°C	7 (21%)	3 (12%)	
Temperature unknown	0 (0%)	0 (0%)	
Complex TGA	14 (41%)	9 (38%)	0.78
Postoperative arrhythmias	3 (9%)	1 (4%)	0.64
Pacemaker before 1990	5 (15%)	4 (17%)	0.22
Follow-up, years	46 [42-46]		
Age at study, years	46 [43-49]		

*Exclusion of the deceased patients

Supplementary Table 2B. Additional information about TGA cohort during last evaluation moment

	2022<br>N=34
Hypertension	2 (7%)
Diabetes Mellitus II	3 (11%)
Hypercholesterolemia	1 (4%)
Smoking	1 (4%)
Medication	
None	9 (10%)
ACE inhibitors/angiotensin receptor neprilysin inhibitors	21 (62%)
Sacubitril/valsartan	3 (9%)
Beta blockers	9 (10%)
Calcium antagonists	1 (1%)
Loop diuretics	5 (15%)
Mineralcorticosteroids receptor antagonists	7 (21%)
$SGLT_2$ inhibitors	4 (12%)
Oral anticoagulants	10 (11%)
Aspirin	0 (0%)
Antiarrhythmics	10 (11%)
Digoxin	0 (0%)
Oral nitrates	0 (0%)
Cholesterol-lowering drugs	2 (2%)

Supplementary Table 3. Details regarding causes of death TGA patients.

Patient (sex/ age at death)*	Follow-up until death (years)	Cause of death
Postoperative		
Patient 1 (♀, 0 years old)	0	Low cardiac output syndrome, autopsy: collapsed lung tissue
Patient 2 (♀, 0 years old)	0	Cardiac tamponade
Patient 3 (♂, 0 years old)	0	Massive bleeding in the trachea
Patient 4 (♂, 0 years old)	0	Circulatory problems and clinical deterioration due to sepsis
Patient 5 (♂, 0 years old)	0	Epileptic seizures and increasing decompensation with clinical worsening; autopsy: bilateral hydrothorax and ascites
Patient 6 (♀, 0 years old)	0	After admission, returned to Portugal, died 16 days after surgery (cause unknown)

Other	Follow-up until death (years)	Simple or complex TGA	Rhythm[†]	PQ time / QRS duration (ms)[†]	RV dysfunction / TR[†]	Arrhythmia on Holter analyses	Cause of death
Patient 7 (♀, 3 years old)	1	Simple	-	-	-	-	Pulmonary edema
Patient 8 (♂, 3 years old)	3	Simple	-	-	-	-	Sudden death
Patient 9 (♂, 5 years old)	1	Complex	-	-	-	-	Congestive heart failure
Patient 10 (♂, 5 years old)	3	Simple	-	-	-	-	Sudden death
Patient 11 (♀, 6 years old)	1	Complex	-	-	-	-	Sudden death
Patient 12 (♀, 7 years old)	6	Complex	-	-	-	-	RV failure
Patient 13 (♂, 8 years old)	1	Complex	-	-	-	-	Unknown
Patient 14 (♂, 8 years old)	5	Simple	-	-	-	-	Sudden death
Patient 15 (♀, 9 years old)	5	Simple	-	-	-	-	Arrhythmia
Patient 16 (♂, 12 years old)	6	Simple	-	-	-	-	Unknown
Patient 17 (♂, 13 years old)	12	Simple	-	-	-	-	Sudden death during exercise
Patient 18 (♂, 16 years old)	12	Simple	-	-	-	-	SVT
Patient 19 (♀, 17 years old)	10	Complex	-	-	-	-	Unknown
Patient 20 (♂, 24 years old)	23	Simple	Sinus	-	Yes / no	No	Sepsis and heart failure
Patient 21 (♂, 25 years old)	24	Simple	Nodal	-	Yes / no	SND	VF most likely caused by severe heart failure
Patient 22 (♀, 33 years old)	26	Simple	Sinus / AFL	-	Yes / no	-	Heart transplantation
Patient 23 (♂, 33 years old)	32	Simple	Sinus	196 / 134	Yes / yes	SND / nsVT	VF during exercise
Patient 24 (♂, 37 years old)	36	Simple	Sinus	235 / 161	Yes / yes	No	Heart transplantation
Patient 25 (♂, 38 years old)	38	Simple	PM	- / 251	Yes / yes	No	End-stage heart failure
Patient 26 (♂, 40 years old)	39	Simple	Sinus	210 / 107	Yes / yes	No	Malignancy
Patient 27 (♂, 41 years old)	36	Simple	Atrial	- / 128	Yes/ yes	SND / SVT	VF after upgrading epicardial PM to biventricular ICD
Patient 28 (♂, 41 years old)	41	Complex	Sinus	233 / 114	Yes / yes	No	End-stage heart failure
Patient 29 (♀, 45 years old)	42	Complex	Sinus	178 / 153	Yes / yes	SVT	Known with end-stage heart failure, chose to end life with euthanasia
Patient 30 (♂, 46 years old)	42	Simple	Sinus	205 / 138	Yes / yes	No	Cardiogenic shock/end-stage heart failure

Patient 31 (♀, 48 years old)	37	Complex	AF	- / 94	Yes / yes	SVT	Electromechanical dissociation after defibrillation threshold testing during ICD implantation
Patient 32 (♀, 50 years old)	44	Simple	AF	- / 136	Yes / yes	AF	Pacemaker change, died after 6 weeks from retroperitoneal hemorrhage
Patient 33 (♀, 51 years old)	43	Complex	PM	- / 192	Yes / yes	AF	Ischemic stroke

AF= atrial fibrillation; AFL= atrial flutter; HF= heart failure; nsVT= non-sustained ventricular tachycardia; PM= pacemaker; SCD= sudden cardiac death; SND= sinus nodal dysfunction; SR= sinus rhythm; sRVEF= systemic right ventricular function; TR= tricuspid regurgitation

*Patients 1-19 died before 1990 (first evaluation moment)

†Results of the last evaluation moment

Supplementary Table 4. Total of first events

First events*	Before 2012	2012-2022	Total
Death or heart transplantation	24	9	33
Reintervention	31	3	34
Baffle intervention	21	2	23
VSD closure	2	0	2
Closure open ductus Botalli	3	0	3
Other	5	1	6
Symptomatic arrhythmias	25	8	33
SVT	21	8	29
VT	7	4	11
Pacemaker	24	3	27
Epicardial	7	1	8
Endocardial	17	2	19
ICD	6	5	11
Symptomatic heart failure	14	10	24
CVA	0	4	4
Endocarditis	1	0	1

CVA= cerebrovascular accident; ICD= implantable cardioverter defibrillator; SVT= supraventricular tachycardia; VSD= ventricular septal defect; VT= ventricular tachycardia

**Some patients had more than 1 event*

Supplementary Table 5. Correlation analyses

Variable	SVT	HF	NT-proBNP
**VT**	p=0.02	p<0.001	r_{pb}=0.5, p=0.005
**HF**	p<0.001		r_{pb}=0.39, p=0.03
**QRS duration**	*ns*	r_{pb}=0.58, p=0.001	*ns*
**Exercise capacity**	*ns*	r_{pb}=-0.47, p=0.03	r=-0.66, p<0.001
**VO_2max**	r_{pb}=-0.44, p=0.04	r=-0.52, p=0.02	r=-0.74, p<0.001
**NT-proBNP**	*ns*	*ns*	
**sRVEF (MRI)**	*ns*	*ns*	r=-0.86, p<0.001
**RVEDV**	*ns*	*ns*	r=0.66, p=0.03
**RVESV**	*ns*	*ns*	r=0.74, p=0.01
**Physical Function**	*ns*	*ns*	r=-0.69, p=0.002
**Vitality**	*ns*	*ns*	r=-0.64, p=0.005
**Social Functioning**	*ns*	*ns*	r=-0.54, p=0.02

Supplementary Table 6. Laboratory results

	**N**	**2022**
Creatinin (umol/L)	29	86.0 [76.0-92.5]
Creatinin abnormal[†]		4 (14%)
NT-proBNP (pmol/L)	**31**	**45.0 [30.0-63.0]**
NT-proBNP ≥15 pmol/L		**30 (97%)**
HS-Troponin T (ng/L)	22	10.0 [6.8-15.5]
HS-Troponin T ≥14 ng/L		**7 (32%)**
Lipid spectrum		
Total cholesterol (mmol/L)	23	4.5 [4.2-5.5]
Total cholesterol abnormal[#]		1 (4%)
LDL cholesterol (mmol/L)	23	3.0 [2.4-3.9]
LDL cholesterol abnormal[$]		**9 (39%)**
HDL cholesterol (mmol/L)	22	1.3 [1.1-1.5]
HDL cholesterol ≤1.55 mmol/L		**19 (86%)**
Triglycerids (mmol/L)	23	1.3 [1.0-1.6]
Triglycerids ≥2 mmol/L		2 (9%)
CRP (mg/L)	21	1.6 [0.7-3.7]
CRP ≥10 mg/L		2 (10%)
Hb (mmol/L)	27	9.3 [8.9-9.8]
Hb abnormal[÷]		2 (7%)
TSH (mU/L)	25	1.7 [1.2-2.2]
TSH abnormal[∆]		2 (8%)
FT4 (pmol/L)	24	19.0 [17.3-20.8]
FT4 abnormal[€]		1 (4%)
HbA1c (mmol/mol)	21	38.0 [35.0-46.0]
HbA1c abnormal[%]		**9 (43%)**

CRP= C-reactive protein; FT4= free thyroxine; HbA1C= glycated haemoglobin; Hb= hemoglobin; HDL= High-density lipoprotein; HS-Troponin-T= high-sensitivity cardiac troponin T; Ht= hematocrit; LDL= low-density lipoprotein; MCV= mean corpuscular volume; NT-proBNP= N-terminal pro b-type natriuretic peptide; RDW= red cell distribution width; TSH= thyroid stimulating hormone

Supplementary Table 7. Additional analysis: predictors of outcome events, using parameters measured in 1990

	Univariable Model			Multivariable Model†		
**End Point***	**HR**	**95% CI**	**P-value**	**HR**	**95% CI**	**P-value**
All-cause mortality (n=33)						
PR time (per 10 milliseconds)	1.03	[0.83-1.28]	0.76			
QRS duration (per 10 milliseconds)	1.39	[0.75-2.60]	0.30			
Definitive pacemaker	1.12	[0.25-5.15]	0.88			
Exercise capacity (per 10%)	1.02	[0.79-1.33]	0.86			
sRVF#	7.51	[1.32-42.72]	**0.02**	7.51	[1.32-42.72]	**0.02**
Composite endpoint (mortality and heart failure) (n=57)						
PR time (per 10 milliseconds)	1.04	[0.94-1.15]	0.44			
QRS duration (per 10 milliseconds)	1.29	[0.88-1.89]	0.20			
Definitive pacemaker	1.71	[0.64-4.58]	0.29			
Exercise capacity (per 10%)	0.99	[0.84-1.17]	0.89			
sRVF#	8.04	[2.43-26.65]	**<0.001**	8.04	[2.43-26.65]	**<0.001**

sRVF= systemic right ventricular function (echocardiography)
*Cox regression
†Adjusted for sex in both univariate and multivariate analyses, the other variables were excluded due to non-significant results
#The variable ''sRVF'' is dichotomous, with 0 representing a normal and 1 representing diminished function

Supplementary Table 8. 36-Item Short Form Survey results of the TGA cohort

		TGA QoL3 (n=31)			TGA QoL4 (n=20)			Norm (n=1742)			P-value QoL4 vs QoL3	P-value QoL4 vs GDP
Physical complex												
Physical Function	RP	85.2	±	13.9	82.8	±	18.2	83.0	±	22.8	0.20	0.95
Role Physical	BP	87.1	±	28.8	75.0	±	37.2	76.4	±	36.3	**0.009**	0.87
Bodily Pain	SF	89.4	±	18.5	84.9	±	19.1	74.9	±	23.4	**0.02**	**0.03**
General Health	PF	61.0	±	15.5	51.8	±	20.0	70.7	±	20.7	**0.01**	**<0.001**
Mental complex												
Vitality	GH	74.8	±	15.8	65.5	±	17.9	68.6	±	19.3	**<0.001**	0.45
Social Functioning	MH	94.6	±	12.6	88.8	±	18.1	84.0	±	22.4	**0.02**	0.26
Mental Health	RE	80.9	±	14.8	80.0	±	15.5	76.8	±	17.4	0.15	0.37
Role Emotional	VT	91.7	±	26.6	90.0	±	26.7	82.3	±	32.9	0.11	0.21

		TGA QoL4 Male (n=13)			TGA QoL4 Female (n=7)			Norm (n=1742)			P-value Male vs Norm	P-value Female vs Norm	P-value Male vs Female
Physical complex													
Physical Function	PF	79.2	±	20.9	89.3	±	9.8	83.0	±	22.8	0.53	0.14	0.16
Role Physical	RP	76.9	±	37.4	71.4	±	39.3	76.4	±	36.3	0.96	0.75	0.77
Bodily Pain	BP	84.0	±	22.1	86.4	±	13.4	74.9	±	23.4	0.16	0.06	0.77
General Health	GH	46.5	±	16.0	61.4	±	24.1	70.7	±	20.7	**<0.001**	0.35	0.18
Mental complex													
Vitality	VT	62.7	±	14.9	70.7	±	22.8	68.6	±	19.3	0.18	0.81	0.42
Social Functioning	SF	89.4	±	20.9	87.5	±	12.5	84.0	±	22.4	0.37	0.49	0.80
Mental Health	MH	81.8	±	12.0	76.6	±	21.3	76.8	±	17.4	0.15	0.98	0.56
Role Emotional	RE	89.7	±	28.5	90.5	±	25.2	82.3	±	32.9	0.37	0.42	0.95

Norm= general Dutch population; QoL3= Quality of Life 3 (2012); QoL4= Quality of Life 4 (2022); TGA= transposition of the Great Arteries

Supplementary Figure 1. Sensitivity analysis of patients who participated in all four evaluation time points

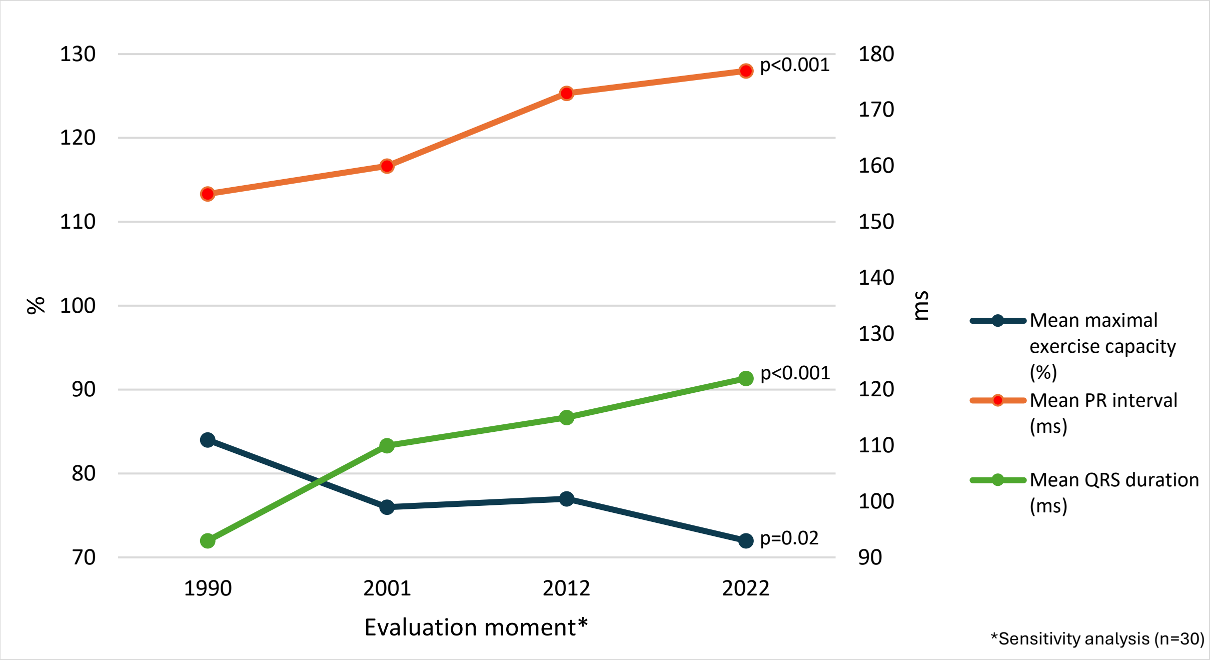

Supplementary Figure 2A. 36-Item Short Form Survey results of the TGA cohort compared to 10 years ago

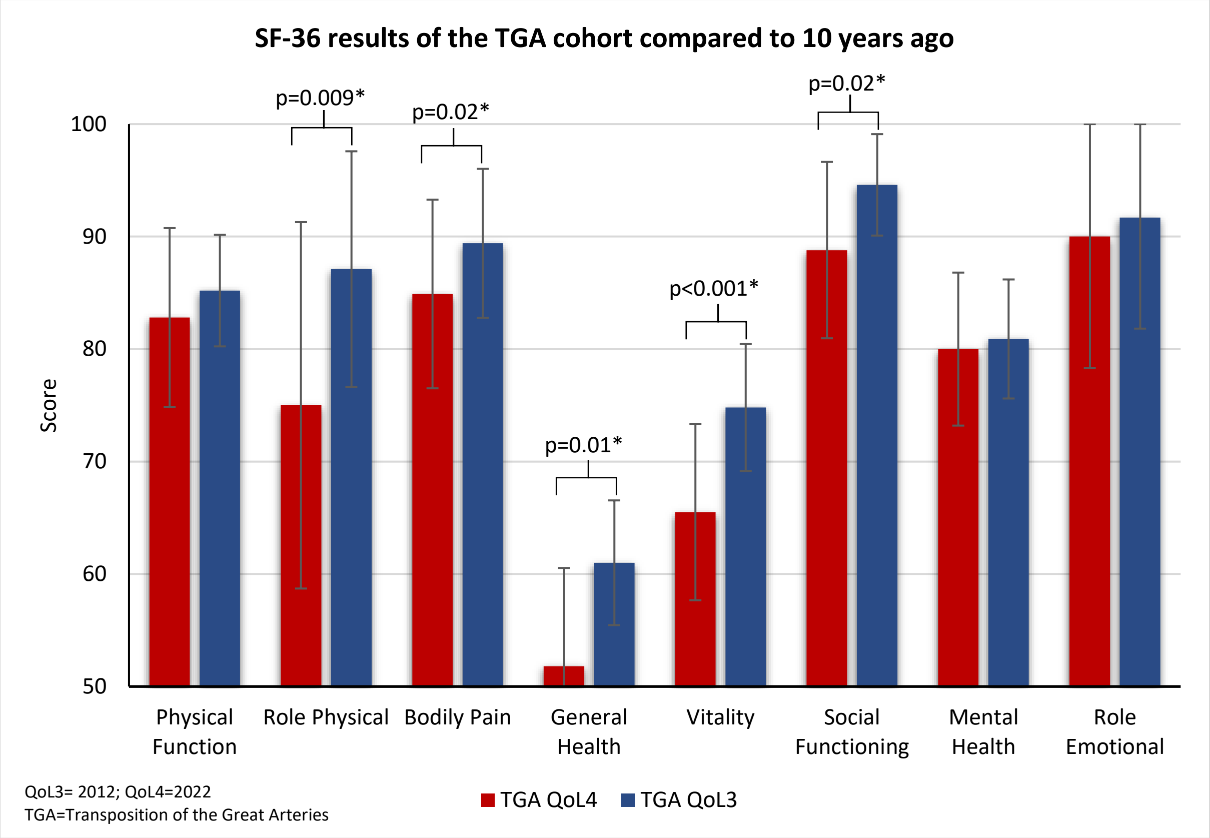

Supplementary Figure 2B. 36-Item Short Form Survey results of the TGA cohort over the years

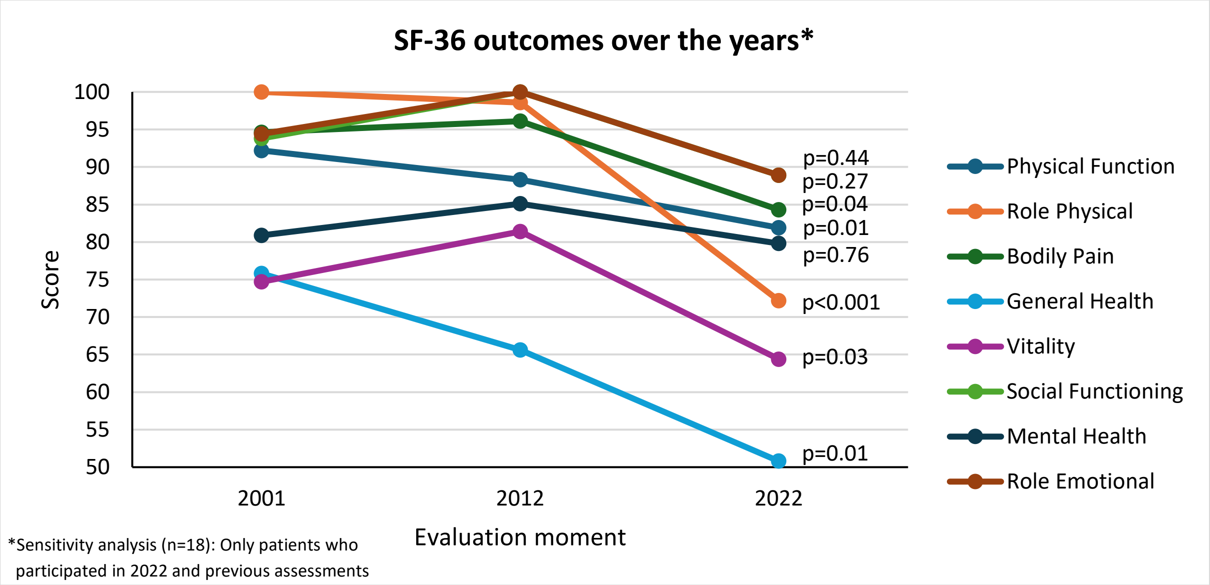

Supplementary Figure 3. 36-Item Short Form Survey results of the male and female TGA patients compared to the general Dutch population

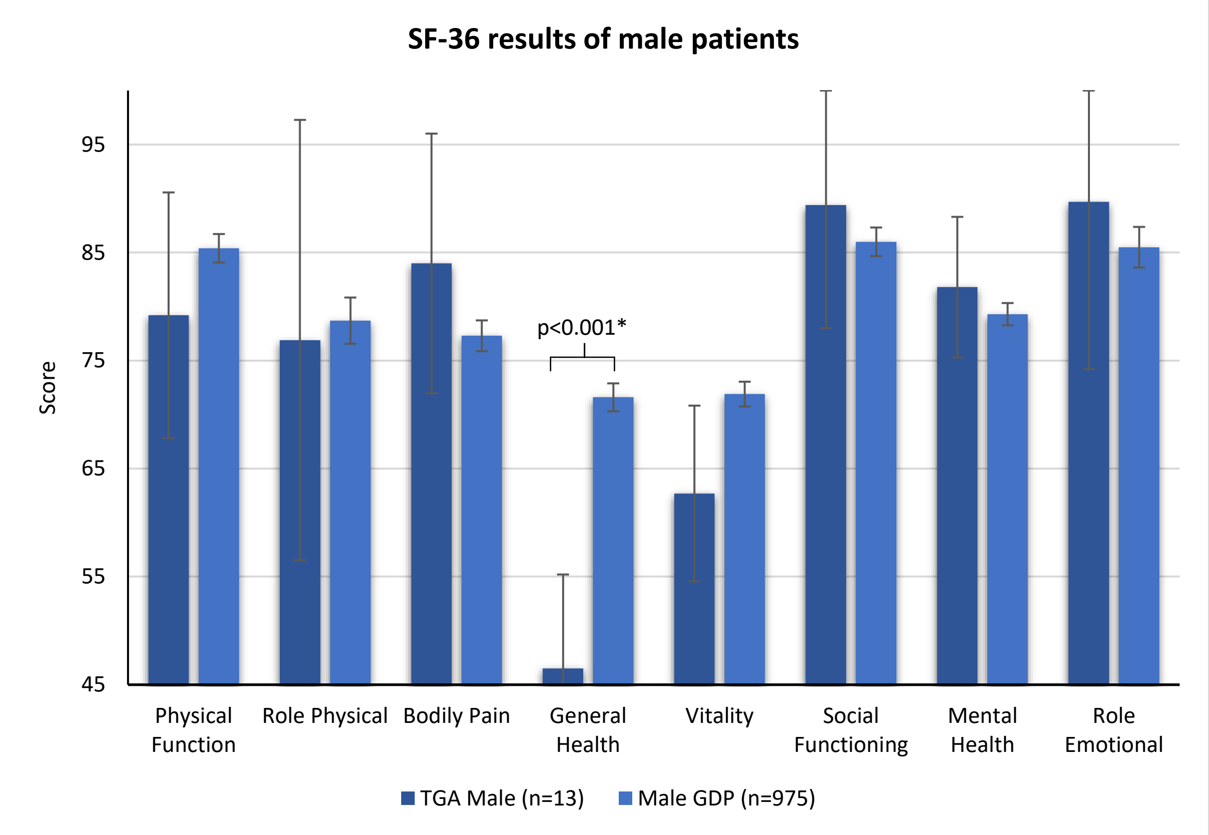

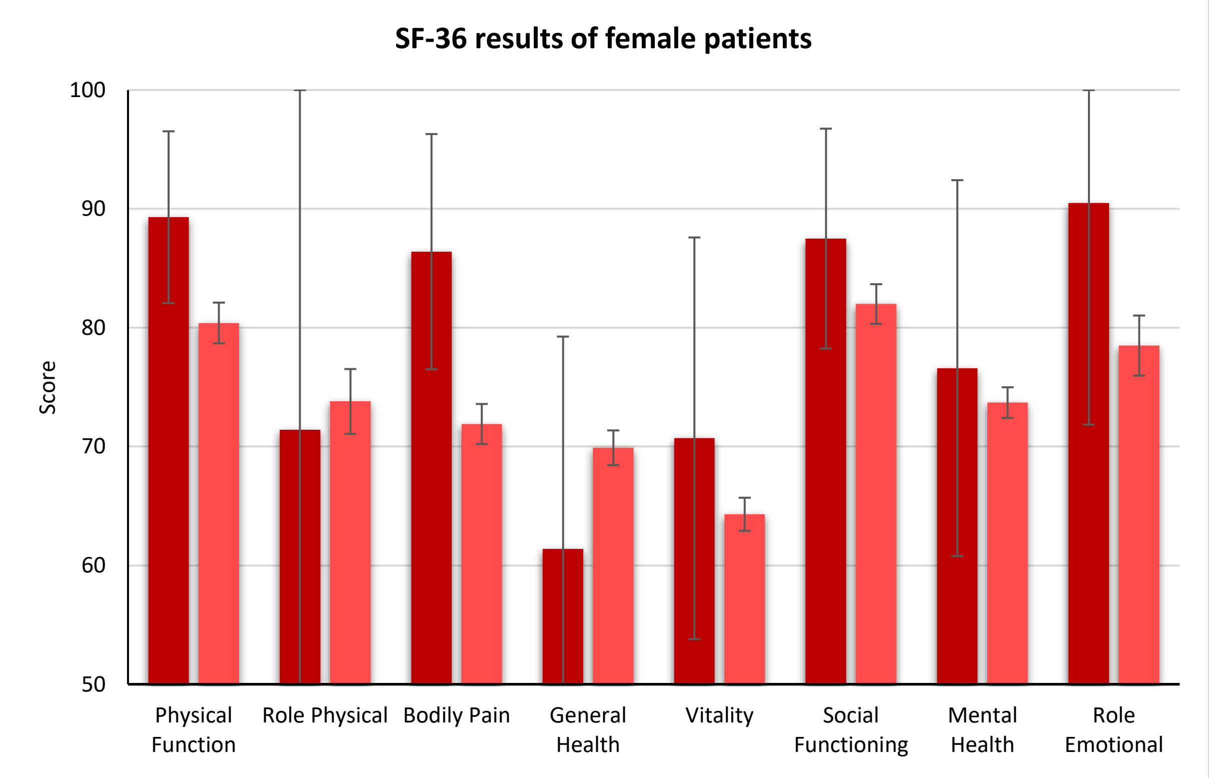

GDP= general Dutch population
TGA= Transposition of the Great Arteries
